# The Neurophysiological Representation of Imagined Somatosensory Percepts in Human Cortex

**DOI:** 10.1523/JNEUROSCI.2460-20.2021

**Published:** 2021-03-10

**Authors:** Luke Bashford, Isabelle Rosenthal, Spencer Kellis, Kelsie Pejsa, Daniel Kramer, Brian Lee, Charles Liu, Richard A. Andersen

**Affiliations:** ^1^Division of Biology and Biological Engineering, California Institute of Technology, Pasadena, California 91125; ^2^T&C Chen Brain-Machine Interface Center, California Institute of Technology, Pasadena, California 91125; ^3^Department of Neurological Surgery, Keck School of Medicine of University of Southern California, Los Angeles, California 90033; ^4^USC Neurorestoration Center, Keck School of Medicine of University of Southern California, Los Angeles, California 90033; ^5^Rancho Los Amigos National Rehabilitation Center, Downey, California 90242

**Keywords:** brain-machine interface, human, intracortical microstimulation, sensation, somatosensation

## Abstract

Intracortical microstimulation (ICMS) in human primary somatosensory cortex (S1) has been used to successfully evoke naturalistic sensations. However, the neurophysiological mechanisms underlying the evoked sensations remain unknown. To understand how specific stimulation parameters elicit certain sensations we must first understand the representation of those sensations in the brain. In this study we record from intracortical microelectrode arrays implanted in S1, premotor cortex, and posterior parietal cortex of a male human participant performing a somatosensory imagery task. The sensations imagined were those previously elicited by ICMS of S1, in the same array of the same participant. In both spike and local field potential recordings, features of the neural signal can be used to classify different imagined sensations. These features are shown to be stable over time. The sensorimotor cortices only encode the imagined sensation during the imagery task, while posterior parietal cortex encodes the sensations starting with cue presentation. These findings demonstrate that different aspects of the sensory experience can be individually decoded from intracortically recorded human neural signals across the cortical sensory network. Activity underlying these unique sensory representations may inform the stimulation parameters for precisely eliciting specific sensations via ICMS in future work.

**SIGNIFICANCE STATEMENT** Electrical stimulation of human cortex is increasingly more common for providing feedback in neural devices. Understanding the relationship between naturally evoked and artificially evoked neurophysiology for the same sensations will be important in advancing such devices. Here, we investigate the neural activity in human primary somatosensory, premotor, and parietal cortices during somatosensory imagery. The sensations imagined were those previously elicited during intracortical microstimulation (ICMS) of the same somatosensory electrode array. We elucidate the neural features during somatosensory imagery that significantly encode different aspects of individual sensations and demonstrate feature stability over almost a year. The correspondence between neurophysiology elicited with or without stimulation for the same sensations will inform methods to deliver more precise feedback through stimulation in the future.

## Introduction

In recent studies, intracortical microstimulation (ICMS) in primary somatosensory cortex (S1) has been successfully used to elicit somatosensory sensations in quadriplegic humans below the level of spinal cord lesion (Flesher et al., 2016; Salas et al., 2018). Many parameters of the electrical stimulus, such as amplitude, frequency, duration, and electrode location, have been found to manipulate the qualitative experience of elicited sensory responses in both non-human primates and humans (Kim et al., 2015a,b; Flesher et al., 2016; Salas et al., 2018; Sombeck and Miller, 2019; Callier et al., 2020). It is therefore important to develop our understanding of the correspondence between stimulation parameters and the sensations they elicit if we are to further understand the mode of action of ICMS and elicit specific sensations more reliably via ICMS. To begin, we seek to uncover the neurophysiology underlying those sensations previously elicited by ICMS.

In previous work (Salas et al., 2018), we found the top five most elicited somatic sensations with ICMS in S1 of a human participant. These were naturalistic sensations which the subject had not experienced in deafferented locations since being injured. We seek to examine for the first time the intracortical electrophysiological behavior of human sensorimotor circuits while experiencing these same sensations. Since it is not possible to use normal touch to elicit a sensation below the level of paralysis in a quadriplegic individual, we performed our experiment using “somatosensory imagery”, the vivid recollection of a somatosensory experience, to evoke activity in these circuits specific to the same sensations experienced during electrical stimulation. We chose to use sensations that were previously elicited by ICMS, rather than any sensation the subject was able to imagine, because these sensations were elicited with known stimulation parameters in the same cortical area we record from during somatosensory imagery.

**Figure 1.**
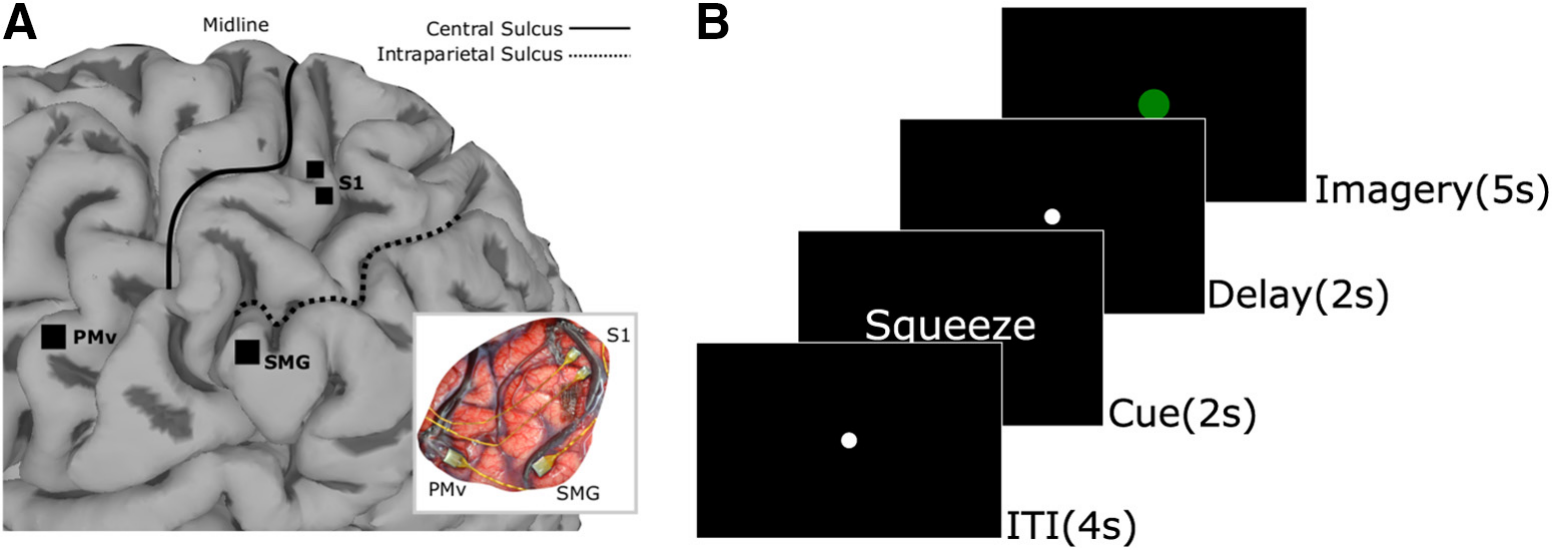
Methods. ***A***, Array implant locations. ***B***, Task paradigm: (1) ITI, 4 s; (2) cue phase displaying the sensation to be imagined, 2 s; (3) delay phase, 2 s; (4) imagery phase during which time the participant recalls as vividly as possible the sensation presented during the cue, 5 s.

Somatosensory imagery has previously been shown in functional magnetic resonance imaging (fMRI) studies to activate the somatosensory system (Hodge et al., 1996; Fitzgibbon et al., 2012). Both primary and secondary somatosensory areas are activated by tactile imagery (Yoo et al., 2003) in areas that respond to actual touch. Imagined movements after amputation of the fingers have also been shown to produce neural activation in somatosensory cortex (Rosén et al., 2001). We record intracortically from three areas of human cortex (Fig. 1*A*), S1, ventral premotor cortex (PMv), and the supramarginal gyrus (SMG). Each of these areas is involved in somatosensory processing. Neurons in S1 respond to cutaneous and proprioceptive stimuli (Hyvärinen and Poranen, 1978; Iwamura et al., 1993; Taoka et al., 2000; Seelke et al., 2012) and electrical stimulation in this area produces naturalistic somatosensory percepts (Flesher et al., 2016; Salas et al., 2018). The SMG array, on the SMG near the anterior end of the intraparietal sulcus (Fig. 1*A*), is in a region of cortex often studied in the context of grasp for both human (Binkofski et al., 1998; Culham et al., 2003) and non-human primate (Dong et al., 1994; Baumann et al., 2009) studies. There is not yet enough evidence in this literature and our study to make exact homological assignments between the two species. Similarly, this same region of cortex responds to somatosensory stimuli in both species (Leinonen et al., 1979; Dong et al., 1994) and has reciprocal connections to other sensorimotor regions such as BA1, BA2, BA5, S2 (Neal et al., 1990), and premotor cortex (Gregoriou et al., 2006). Broadly, posterior parietal cortex is a higher order area in sensorimotor and somatosensory processing (Romo et al., 1998; Romo and de Lafuente, 2013; Aflalo et al., 2015). PMv neurons respond to tactile and proprioceptive somatosensory stimuli (Fogassi et al., 1996; Graziano et al., 1997; Graziano, 1999). Given the role of these areas in somatosensory processing, we expect to observe neurophysiological modulation because of somatosensory imagery.

In this work we investigated the neural correlates of imagined sensations and how this representation is distributed across different sensorimotor cortical areas. We used the sensations previously experienced by our participant during ICMS (Salas et al., 2018) and sought to demonstrate a discriminable representation of the sensations in the brain. We examined neurophysiological responses to somatosensory imagery from intracortical human recordings across three brain areas, each implanted with recording microelectrode arrays (Utah Array, Blackrock Microsystems): S1, SMG, and PMv. We found a highly significant classification accuracy between sensations was attainable using both threshold crossing spiking activity and spectral power of various common frequency bands in the continuous brain signal.

Our results demonstrate that unique sensory experiences can be classified from human neural signals during somatosensory imagery and explore how the encoding of different aspects of sensation are distributed across different brain areas. The correspondence between the neural signal during somatosensory imagery and the stimulation parameters that elicit the same sensations may inform the choice of stimulation parameters for eliciting novel and robust sensations via ICMS in future work.

## Materials and Methods

### 

#### Participant

We recruited and consented a male participant with C5-level complete spinal cord injury (34 years old, three years and six months postinjury, and one year and eight months postimplant, at the time of the first experiment) to participate in a clinical trial of a brain-machine interface (BMI) system with intracortical recording and stimulation. All data were recorded through electrode arrays that were implanted in three locations of the left hemisphere (Fig. 1*A*): SMG, PMv, and S1. One 96-channel, platinum tipped Neuroport microelectrode recording array (Blackrock Microsystems, Salt Lake City, UT) was implanted in each of SMG and PMv. Two 48-channel SIROF-tipped (sputtered iridium oxide film) microelectrode arrays were implanted in S1. Further information regarding specific surgical planning and implantation details are described in (Salas et al., 2018). All procedures were approved by the Institutional Review Boards (IRB) of the California Institute of Technology, University of Southern California, and Rancho Los Amigos National Rehabilitation Hospital.

#### Task

Based on the outcome of S1-only stimulation mapping we identified the five most commonly elicited sensations with ICMS: “squeeze,” “tap,” “rightward movement,” “vibration,” and “blowing.” These sensations represented 24.9%, 17.3%, 9.7%, 8.1%, and 6.6%, respectively, of 381 total ICMS elicited sensations (for full details of ICMS mapping see, Salas et al., 2018). These sensations were experienced in the same body locations of the contralateral forearm and upper arm. In our somatosensory imagery experiment, each trial consisted of an intertrial interval (ITI), a cue, a delay, and an imagery phase. During the ITI, a black screen with a gray circle (1-cm diameter) in the middle was shown for 4 s during which time the participant was instructed to rest and fixate gaze on the circle, although gaze was not measured. In the cue phase, one of the sensations listed above was presented as a written word for 2 s, then in the 2-s delay phase, only a black screen with the fixation circle was shown. In the final 5-s imagery phase of the task, the fixation circle changed to green and the participant began somatosensory imagery. The instruction for the imagery phase given at the beginning of each experiment was to “imagine the sensation as you experienced it during electrical stimulation as vividly as possible” (Fig. 1*B*). The participant confirmed to us that the sensations were all imagined in the same location at the forearm, thus controlling for the inadvertent classification of location rather than sensation. In each run of the task, each individual sensation was imagined 10 times (total 50 trials per run), pseudo-randomly shuffled. The full dataset consists of 400 trials with *N* = 80 repetitions of each imagined sensation.

#### Experiment design and data collection

Data were collected from each array site using a 128-channel Neural Signal Processor (Blackrock Microsystems). Broadband signals were recorded at 30,000 samples/s. Spectral power was computed for each phase of each trial using MATLAB's pspectrum function (MathWorks Inc. MA). Unsorted threshold crossings (Christie et al., 2015; Oby et al., 2016; Dai et al., 2019) extracted from the broadband signal using a threshold of −3.5 times the noise RMS of the continuous signal voltage, were used as spike activity. The first full data set (herein referred to as experiment 1) was collected across 10 d. The second full data set was collected 11 months later, across 24 d (herein referred to as experiment 2). This time delay allowed us to explore the stability of the representations initially observed. ICMS sensory mapping (Salas et al., 2018) that produced the percepts used for imagery in this study were collected 16 months before experiment 1 began.

#### Statistics and analysis methods

Classification was performed independently for each array and each phase of the somatosensory-imagery task using linear discriminant analysis (LDA) with the fitcdiscr function in MATLAB. For analysis using spike firing rates, the average threshold crossing rates from each channel, calculated from the entirety of each phase in 50-ms time bins, were passed as features to the classifier. For analysis of the spectral power data, power in the 4–8 (θ), 8–12 (α), 12–30 (β), 30–70, 70–150, and 150–300 Hz (γ) bands, computed for each channel, were used as features. Classification was performed separately for each frequency band. We note that in these very high-frequency bands the signal is likely to reflect the spiking activity of local neurons.

For both threshold crossings and spectral power, LDA was performed over 1000 repetitions. In each repetition, all 400 trials were randomly divided in a 50/50 cross-validation training and testing paradigm. Following 1000 repetitions, mean classification accuracy and 95% confidence intervals were computed. This procedure was repeated in a null condition where class labels were randomly shuffled during each repetition to generate a chance-level distribution of classification accuracies. Significance for classification performance was calculated by comparison of the overlapping percentile values of the actual and null data set. The full results are available in Table 1.

**Table 1.** Classification accuracy and significance

		SMG			PMv			S1			Dimensions
		Cue	Delay	Imagery	Cue	Delay	Imagery	Cue	Delay	Imagery	
Experiment 1	Spikes	78%***	69%***	82%***	24%, n/s	30%, n/s	37%**	18%, n/s	23%, n/s	38%***	35
	4–8 Hz	33%*	22%, n/s	24%, n/s	23%, n/s	21%, n/s	24%, n/s	19%, n/s	19%, n/s	20%, n/s	45
	8–12 Hz	32%, n/s	21%, n/s	34%**	20%, n/s	22%, n/s	33%*	19%, n/s	22%, n/s	20%, n/s	20
	12–30 Hz	31%*	21%, n/s	28%, n/s	17%, n/s	16%, n/s	27%, n/s	17%, n/s	20%, n/s	23%, n/s	15
	30–70 Hz	35%**	27%, n/s	60%***	18%, n/s	19%, n/s	24%, n/s	16%, n/s	17%, n/s	27%, n/s	30
	70–150 Hz	57%***	44%***	75%***	20%, n/s	22%, n/s	28%, n/s	19%, n/s	22%, n/s	28%, n/s	40
	150–300 Hz	63%***	46%***	77%***	22%, n/s	21%, n/s	32%*	19%, n/s	20%, n/s	34%*	45
Experiment 2	Spikes	92%***	62%***	75%***	27%, n/s	35%**	54%***	21%, n/s	20%, n/s	31%, n/s	35
	4–8 Hz	34%*	24%, n/s	25%, n/s	21%, n/s	20%, n/s	25%, n/s	18%, n/s	18%, n/s	21%, n/s	45
	8–12 Hz	37%**	23%, n/s	27%, n/s	20%, n/s	22%, n/s	32%*	18%, n/s	19%, n/s	20%, n/s	20
	12–30 Hz	31%*	20%, n/s	30%, n/s	20%, n/s	18%, n/s	25%, n/s	18%, n/s	17%, n/s	21%, n/s	15
	30–70 Hz	36%**	28%, n/s	53%***	24%, n/s	26%, n/s	38%**	17%, n/s	16%**	20%, n/s	30
	70–150 Hz	62%***	39%***	71%***	35%*	30%, n/s	51%***	19%, n/s	19%, n/s	25%, n/s	40
	150–300 Hz	64%***	43%***	69%***	31%, n/s	28%, n/s	52%***	21%, n/s	20%, n/s	25%, n/s	45
Combined 1 and 2	Spikes	75%***	59%***	73%***	23%, n/s	27%, n/s	31%**	19%, n/s	21%, n/s	33%***	60
	4–8 Hz	31%**	22%, n/s	24%, n/s	19%, n/s	20%, n/s	27%, n/s	19%, n/s	18%, n/s	19%, n/s	10
	8–12 Hz	31%**	21%, n/s	32%***	20%, n/s	21%, n/s	31%**	18%, n/s	21%, n/s	20%, n/s	25
	12–30 Hz	30%*	20%, n/s	29%*	19%, n/s	18%, n/s	26%, n/s	17%, n/s	19%, n/s	21%, n/s	25
	30–70 Hz	35%***	28%, n/s	52%***	21%, n/s	21%, n/s	30%*	17%, n/s	17%, n/s	22%, n/s	75
	70–150 Hz	53%***	37%***	68%***	24%, n/s	23%, n/s	36%***	20%, n/s	20%, n/s	27%, n/s	75
	150–300 Hz	57%***	37%***	67%***	23%, n/s	22%, n/s	37%***	19%, n/s	19%, n/s	29%*	60
2 trained on 1	Spikes	21%, n/s	23%, n/s	30%***	19%, n/s	23%, n/s	16%, n/s	20%, n/s	17%, n/s	21%, n/s	35
	4–8 Hz	24%, n/s	20%, n/s	20%, n/s	23%, n/s	18%, n/s	18%, n/s	21%, n/s	19%, n/s	22%, n/s	45
	8–12 Hz	27%***	21%, n/s	29%***	22%, n/s	23%, n/s	26%***	21%, n/s	22%, n/s	25%*	20
	12–30 Hz	32%***	20%, n/s	26%***	17%, n/s	20%, n/s	22%, n/s	21%, n/s	23%, n/s	22%, n/s	15
	30–70 Hz	27%***	22%, n/s	24%*	24%**	19%, n/s	24%***	20%, n/s	19%, n/s	21%, n/s	30
	70–150 Hz	24%*	30%***	20%, n/s	20%, n/s	23%, n/s	21%, n/s	18%, n/s	20%, n/s	21%, n/s	40
	150–300 Hz	27%***	22%, n/s	22%, n/s	21%, n/s	21%, n/s	20%, n/s	19%, n/s	19%, n/s	20%, n/s	45
1 trained on 2	Spikes	29%***	33%***	35%***	19%, n/s	23%, n/s	23%, n/s	18%, n/s	18%, n/s	21%, n/s	35
	4–8 Hz	26%**	20%, n/s	21%, n/s	22%, n/s	23%, n/s	26%**	22%, n/s	20%, n/s	24%*	45
	8–12 Hz	27***	22%, n/s	31%***	22%, n/s	23%, n/s	29%***	18%, n/s	22%, n/s	22%, n/s	20
	12–30 Hz	23%, n/s	21%, n/s	23%, n/s	21%, n/s	21%, n/s	23%, n/s	19%, n/s	21%, n/s	21%, n/s	15
	30–70 Hz	26%**	26%**	25%**	23%, n/s	19%, n/s	22%, n/s	20%, n/s	21%, n/s	19%, n/s	30
	70–150 Hz	20%, n/s	23%, n/s	20%, n/s	22%, n/s	23%**	20%, n/s	19%, n/s	18%, n/s	18%, n/s	40
	150–300 Hz	24%*	21%, n/s	29%***	20%, n/s	22%, n/s	19%, n/s	23%, n/s	19%, n/s	21%, n/s	45

Table showing the classification accuracy as a percentage and significance (n/s = not significant, *p < 0.05, **p < 0.01, ***p < 0.001) from each of the experiments in which the five sensations tested were classified with LDA and SVD. Classification was performed separately for each data type (spike or spectral power band), each trial phase (cue, delay, and imagery), and for each brain area (SMG, PMv, and S1). The number of features used for each classification is listed in the dimensions column (see Materials and Methods).

In order to test the ability of our datasets to generalize to one another, a decoder was trained on all of experiment 1 data and tested on all of experiment 2 data (another decoder was trained using the opposite train/test regime). This analysis yielded only one accuracy for each phase and electrode array as opposed to a distribution over 1000 iterations, because of the nature of testing which used one specific split of the data. However, the null condition was calculated as before, by shuffling the trial labels of both train and test datasets randomly over 1000 iterations. For this reason, in the generalization analysis, for each phase and electrode array, a single accuracy value was compared with the percentiles of the null distribution. For instance, we report *p* < 0.05 if the accuracy was greater than the value at the 97.5th percentile of the null distribution.

Initially we performed LDA without preprocessing (e.g., without performing dimensionality reduction) as this allows for a direct analysis of the relationship between the neural activity recorded on each channel and imagined sensations. However, since the absence of preprocessing results in a small trade-off in classification accuracy, we separately repeated the classification using singular value decomposition (SVD) feature selection before model fitting. For threshold-crossing features, SVD was computed on mean-centered firing rates averaged within each task phase (svd function in MATLAB). Average firing rate data were projected onto the top *N* features that represent the dimensions of greatest variance in the data. *N* was determined by examining accuracy scores across phases and electrode arrays in experiment 1. *N* was calculated separately for spike decoding and for each frequency band in spectral power decoding. *N* was initially set to 5 features, and then increased in increments of 5. Each run yielded a mean accuracy across phases (cue, delay, imagery) and arrays (SMG, PMv, S1) over 1000 iterations. For each of these accuracies, the current run was compared with the previous run with *N*–5 features. In all cases, accuracies as N increased followed a curve with a single peak or plateau at some *N* > 0 and smaller than the original number of features. The run with the greater number of superior accuracies was chosen as the “better” run. In the case of a tie, the lower number of features was chosen. The number of features *N* is given in Table 1, dimensions. The number of features determined to be best for experiment 1 data were also used to decode experiment 2 data, and to perform the cross-experiment decoding (i.e., training on experiment 1 and testing on experiment 2 and vice versa). The best number of features was recomputed with the combined data from experiment 1 and experiment 2 following the same procedure. For spectral-power classification, the same approach was used to determine the optimal number of features for each frequency band individually.

As appropriate, *p* values were corrected for multiple comparisons using the Bonferroni–Holm method.

## Results

In this study, a human quadriplegic participant with intracortical microelectrode arrays in the SMG, PMv, and S1 performed somatosensory imagery, the vivid recollection of sensory experiences, of five sensations. These sensations were the most common ones that the same participant experienced in a previously published sensory mapping of S1 by ICMS (Salas et al., 2018). We investigated the hypothesis that somatosensory imagery would generate unique representations for each sensation, which could be classified from the neural signal.

### Classifying sensations

**Figure 2.**
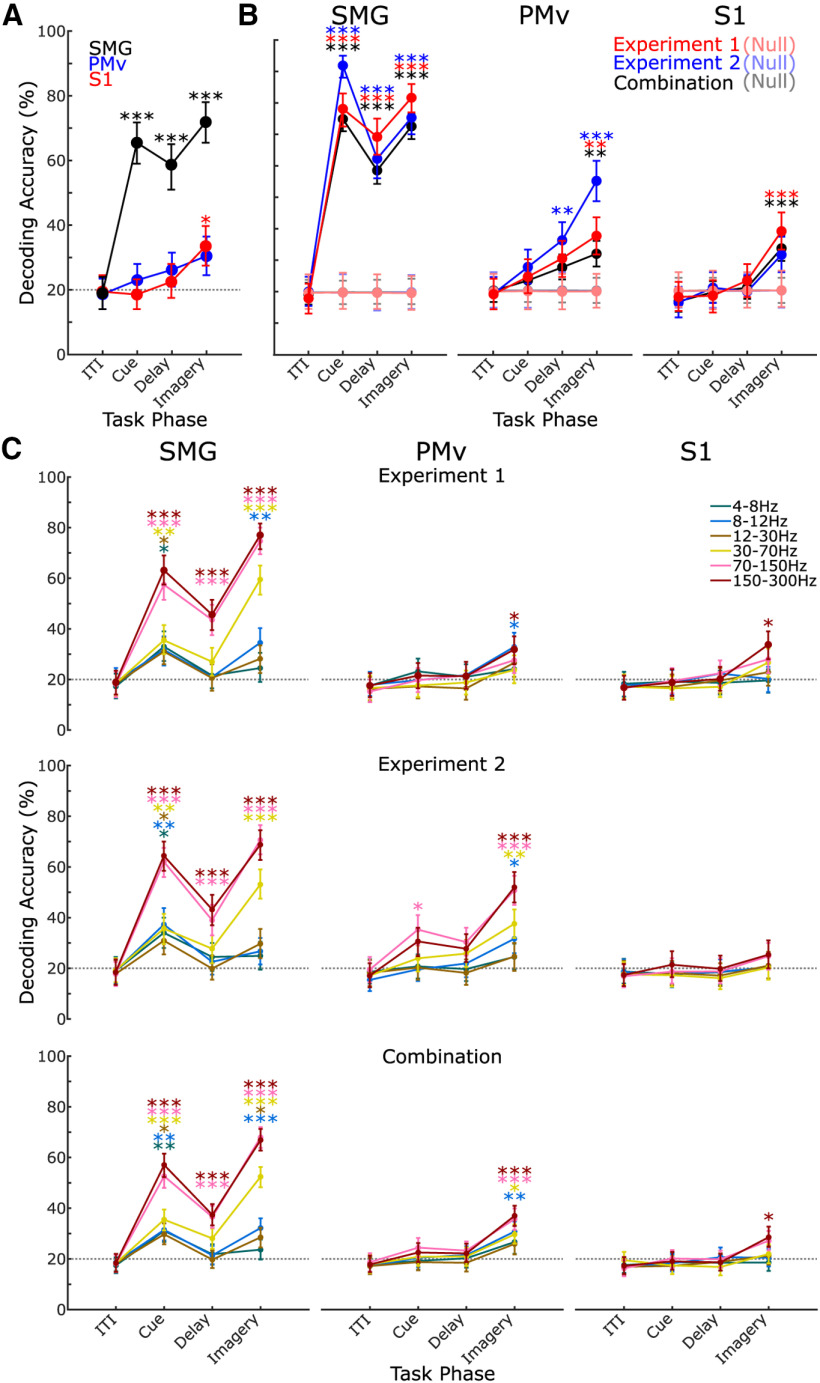
Sensation classification. ***A***, Classification accuracy of sensations with LDA using the spike activity on all channels as features from experiment 1. ***B***, Improved classification accuracy when classifying the sensations using LDA with the spike activity and SVD feature selection from experiment 1 (red), experiment 2 (blue), and the combined experiments 1 and 2 data (black). Each with their own null distribution. ***C***, Classification using spectral power in different frequency bands for experiment 1 (top row), experiment 2 (middle row), and combined experiments 1 and 2 (bottom row). In all subplots, error bars show 95% confidence interval, asterisks denote classification significantly above null distribution. Gray dotted line shows the classification chance level.

**Figure 3.**
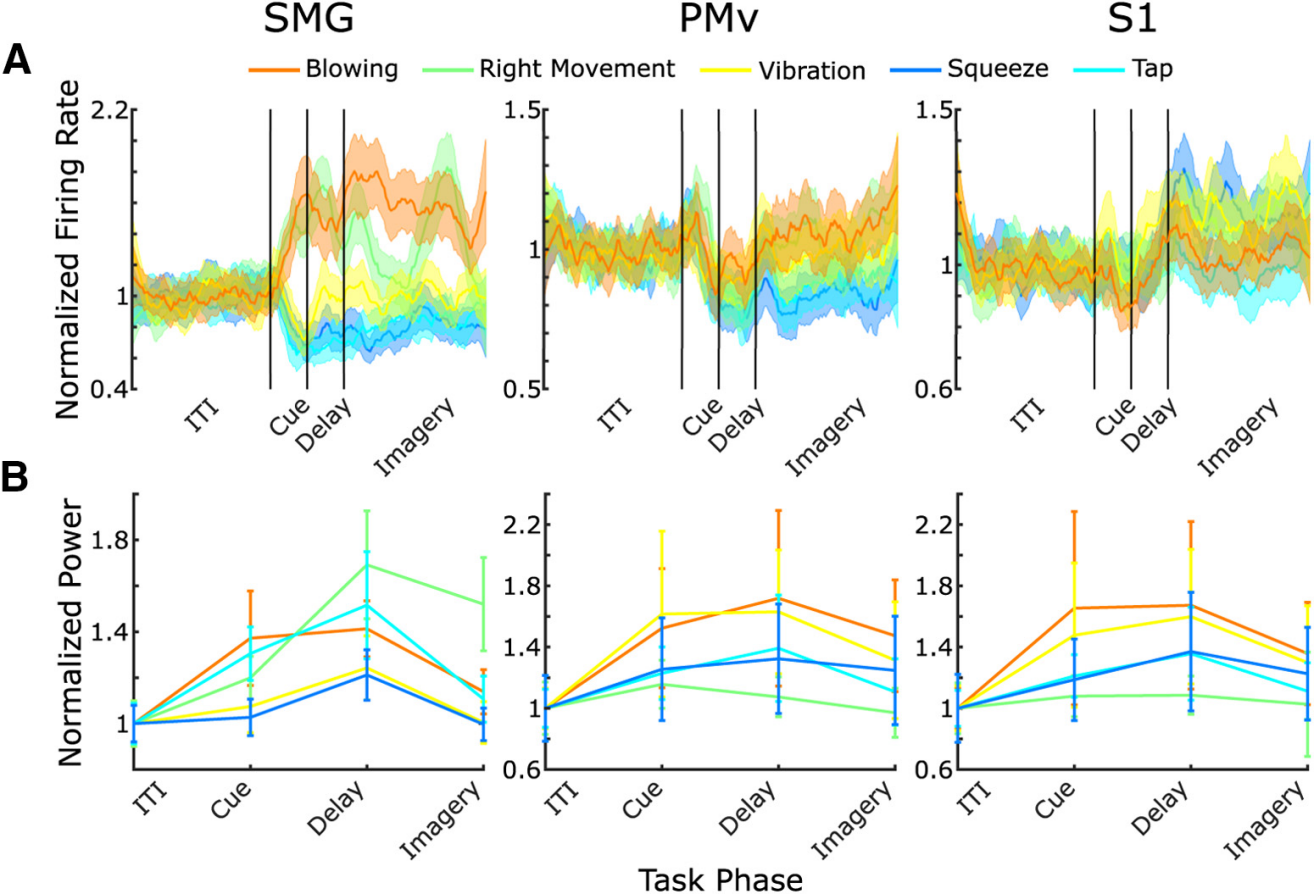
***A***, Mean firing rates across all trials for each sensation on an exemplary channel in each recording location. ***B***, Mean power in the 150- to 300-Hz LFP band across all trials for each sensation on an exemplary channel in each recording locations. Channels show significantly different activity for multiple individual sensations. In all subplots, error bars show 95% confidence interval.

Using unsorted threshold crossings recorded during experiment 1 (see Materials and Methods), we trained an LDA classifier to identify the five sensations we tested. We trained the classifier on half of the trials (see Materials and Methods) at a single phase of the task and on data from a single array, using the average firing rate during the phase at each channel as features. We tested the classification on the other half of the trials in the same phase and array. We found a significant classification accuracy for the cue, delay and imagery phases of the task in SMG and in the imagery phase in S1 (Fig. 2*A*; Table 1). To improve the classification accuracy, we applied SVD feature preprocessing before the LDA was trained (see Materials and Methods). We found significant classification for the cue, delay and imagery phases of the task in SMG and in the imagery phase in both S1 and PMv (Fig. 2*B*, experiment 1; Table 1). In all cases, classification accuracy was compared with that of a null distribution (Fig. 2*B*, null), where the classification was performed identically but the trial labels were randomly shuffled. LDA analysis determines discriminability across the population activity of the whole array; however, we also observed individual channel firing activity capable of significantly discriminating between two or more sensations (exemplary channels shown in Fig. 3*A*). The total percentage of channels, 96 in each brain area, whose activity significantly discriminated between two or more sensations in the imagery phase only (*p* < 0.05) was 49% in SMG, 22% in PMv, and 20% in S1. This metric was calculated per channel, pooling across all trials, using a Kruskal–Wallis test with the averaged firing rate in the imagery phase of the task. Data were corrected for multiple comparisons with the Bonferroni-Holm method. To compare the correspondence between results from both stimulation (in previous work) and imagery for all individual channel-sensation pairs (96 channels × 5 sensations, *N* = 480), we identified tuning of the channel to the sensation by looking for a significant difference in firing rate across all trials of a pair between the ITI and imagery phase of the task, using a Wilcoxon signed rank test (*p* < 0.05). We identified responses to ICMS for each channel-sensation pair by looking for at least one instance of the pair during ICMS mapping in the previous study (Salas et al., 2018). We found 89 (18.5%) pairs (38/96 unique channels, 5/5 unique sensations) which had both neurophysiological tuning and a response to ICMS.

We also used the same method as above to perform a classification using the spectral power in various frequency bands of the raw neural signal as features (see Materials and Methods; Fig. 2*C*; Table 1). In SMG, we found significant classification accuracy in the cue phase across several frequency bands. We also found significant classification accuracy in the delay phase in higher frequency bands only. In the imagery phase we saw significant classification accuracy across several frequency bands. In PMv, we found significant classification accuracy only in the imagery phase at high and low frequencies. Likewise, in S1, we found significant classification accuracy only in the imagery phase and only in the highest frequency band (150–300 Hz).

During the ITI phase of the trial, while the subject was at rest, we never achieved classification performance different to chance level with any method or neural signal used. This confirms that the discriminable activity in other task phases is related specifically to the somatosensory imagery task.

### Longitudinal representation of sensations

We have demonstrated above that different sensations can be uniquely represented in distributed cortical areas. However, to what extent are the representations stable over time? Recordings of the human neural signal can be unstable over time (Aflalo et al., 2015), so to assess longitudinal stability, the participant performed experiment 2, repeating the imagery task ∼11 months after the initial experiment 1. We found that sensations could be classified from threshold crossings in SMG during cue, delay, and imagery phases as in the earlier data (Fig. 2*B*; Table 1). We found a significant classification in the delay and imagery phase in PMv. Using spectral power features from experiment 2 only to examine longitudinal stability, as above with threshold crossings, showed a similar trend. In SMG significant classification was observed in all frequency bands during the cue but in the delay phase was only observed in higher frequency bands (Fig. 2*C*, middle row; Table 1). Additional lower bands became significant in the imagery phase. In PMv, significant classification accuracy was only achieved in the cue phase at a single high-frequency band and in the imagery phase across a range of bands. No significant classification using spectral power was achieved in S1 during experiment 2.

**Figure 4.**
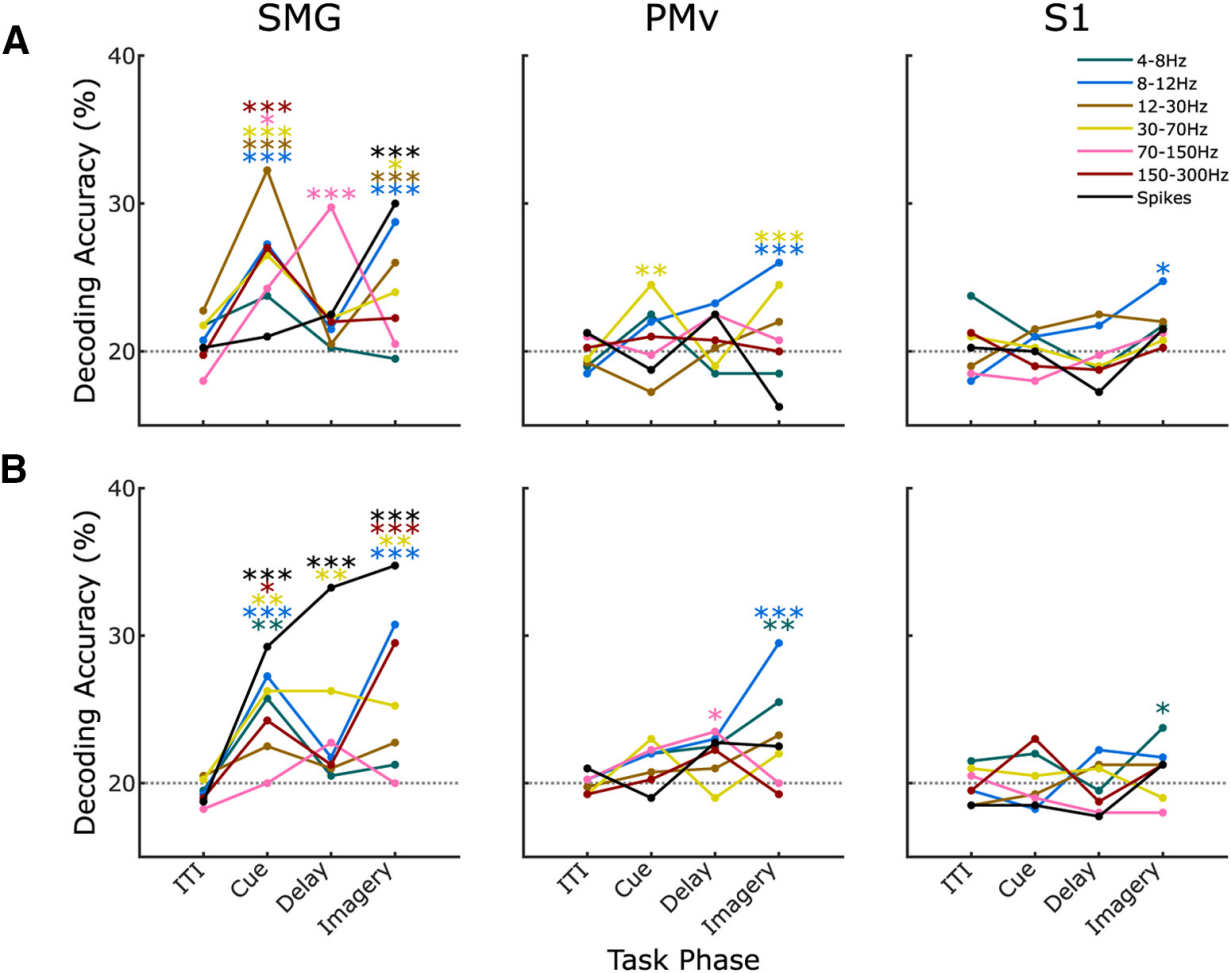
Longitudinal decoding. Classification accuracy calculated per phase and per brain region. ***A***, An LDA with SVD model was trained on all trials in experiment 1 and tested on all trials in experiment 2. ***B***, The same method was used to train on all trials in experiment 2 and test on all trials in experiment 1. Different colors indicate different frequency bands. Gray dotted line shows the classification chance level. Stars indicate significance calculated with respect to the null distribution (see Materials and Methods).

To determine how similar activity was between experiments 1 and 2 within each task phase and each array, we performed a split training and testing using all trials of experiment 1 to train and all trials of experiment 2 to test (and vice versa). A null distribution was created using shuffled labels over *N* = 1000 repetitions of the classification. Using threshold crossings, significant classification accuracy was only observed in SMG during the imagery phase when testing on experiment 2. When testing on experiment 1, significant classification accuracy was observed only in SMG during the cue, delay and imagery phases of the task (Fig. 4).

To evaluate the longitudinal stability of spectral power representations, we trained and tested on both the experiments 1 and 2 datasets, as described above for threshold crossing features. When training on experiment 1 and testing on experiment 2, SMG showed significant classification accuracy in the cue and imagery phase across a broad range of bands and significance in one band in the delay phase. In PMv, significant classification accuracy also occurred in the cue and imagery phase. In S1, significant classification accuracy was observed in the imagery phase at only a low-frequency band (Fig. 4*A*). When training on experiment 2 and testing on experiment 1, significant classification accuracy was observed in SMG during the cue, delay and imagery phase. In PMv, significant classification accuracy occurred during the delay and imagery phase. In S1, significant classification accuracy only occurred during the imagery phase (Fig. 4*B*; Table 1). Longitudinal classification from both spike and LFP signals performs well especially where the signal has a high decoding accuracy within either experiment 1 or 2 alone. In the longitudinal analysis, taking all brain regions together, there are some additional task phases and array locations with significant classification accuracy in the spectral power data compared with the spike data. This indicates a tendency toward more general stability in the spectral power.

To ensure that classification accuracy could not be further improved with more data we combined threshold crossing datasets from both experiment 1 and experiment 2 to use all trials recorded (*N* = 800) in the same classifier, with the same LDA and SVD method as before. Note, data across the two experiments is combined in this model. The significant classification accuracies in this result corroborate stability over time as in the longitudinal analysis above. However, combining data does not take into consideration changes in signal or noise over time as addressed specifically in the longitudinal analysis above. This analysis yielded significant classification accuracy for the cue, delay, and imagery phases of the task in SMG, in the imagery phase in S1 and in the imagery phase of PMv (Fig. 2*B*, combined; Table 1). Finally, we combined the full spectral power data set as above (Fig. 2*C*, bottom row) and found significant classification accuracy for SMG in the cue phase across a broad range of frequency bands, in the delay phase in only the higher frequency bands and imagery phase again across a broad range of bands. In PMv and S1, significant classification accuracy was achieved in the imagery phase only. For PMv, this was achieved in a broad range of frequency bands, while for S1, this was only achieved in the highest frequency band.

## Discussion

As cortical stimulation methods are becoming more widely used it is increasingly important to understand the relationship between intervention (i.e., ICMS) and evoked perception/behavior (i.e., sensations). In order to achieve the goal of restoring sensation in humans, we need to produce consistent effects across participants and robustly deliver specific sensations relevant to the task. We believe understanding this begins with exploring the neural representation of the sensations that we are able to elicit, for example, uncovering the neural features that represent unique sensations. In the work presented here, we demonstrate that different sensations are uniquely represented in the neural activity of human cortex. We measured spiking activity and spectral power during somatosensory imagery with intracortical recording arrays in SMG, PMv, and S1 of a single human participant with a high-level spinal cord injury (see Materials and Methods). We demonstrate that individual sensations can be accurately classified using these signals (Fig. 2; Table 1).

Here, we observe activity through somatosensory imagery, a powerful tool to elicit sensation-relevant neural activity, as physical interaction with the environment is not possible because of the nature of the injury in the quadriplegic patient population. We explore sensations that the participant had experienced both naturally before the injury and reported during ICMS mapping. Previously, individual aspects of somatosensation have been studied in isolation such as responses to different textures, the frequency of vibration, individual forces, etc. In somatosensory imagery, all these components are combined as a naturalistic sensation. With recordings across human cortical areas we can further characterize the distributed response in the brain to somatosensation (Delhaye et al., 2018). We show sensations can be classified in S1 during somatosensory imagery with threshold crossing activity, when the participant vividly recalls a previously experienced sensation (Fig. 2*A*; Table 1). Additionally, in S1 the sensations are only classifiable in the imagery phase in high-frequency spectral power of 150–300 Hz, again likely reflecting spiking activity (Fig. 2*C*, top row; Table 1). This finding suggests S1 does not encode the planning or anticipation of sensation during imagery with no significant classification occurring in the cue or delay phase. In PMv, we found activity in the imagery phase similar to S1, but with additional low-frequency components of 4–8 and 8–12 Hz, which may be responsible for driving coordinated networks over a larger area (Canolty et al., 2006). In experiment 2, we were able to classify sensations from threshold crossing activity in PMv during the delay phase. This result reinforces the trend seen in experiment 1 for PMv (Fig. 2*B*), suggesting that it encodes the planning or anticipation of the sensation in addition to the sensation itself (Fogassi et al., 1996). In SMG, we saw the highest classification performance of any area tested during the cue, delay and imagery phases of the task, both in threshold crossing activity and the spectral power in high-frequency bands. This finding demonstrates SMG contains somatosensory information, both during imagery and in the planning/anticipation of somatosensory imagery (Delhaye et al., 2018). Classification during the cue phase, which uniquely included θ band activity, suggests a representation of the semantic aspect of the cued sensation within SMG, which further supports the higher order cognitive encoding of sensorimotor control in posterior parietal cortex (Andersen and Buneo, 2002; Aflalo et al., 2015; Zhang et al., 2017). We observe a large difference in the decoding performance between SMG and S1/PMv. A hypothesis for this difference may be that since somatosensory imagery is a top-down cognitive process, without somatosensory input, the representation is stronger in SMG as this is a higher order, cognitive area in somatosensory processing. Our results show imagery produces discriminable activity in S1 and PMv; however, the reduced decoding accuracy may reflect the primary role of this neural population to process input from the somatosensory system.

We explicitly test somatosensory imagery to determine whether neural activity encodes the imagined sensation. This is motivated entirely because of the nature of injury in our patient population. We do not assume that these areas would represent the sensations in exactly the same way if they were experienced through interaction with the environment in the absence of injury. Indeed, the representations found from somatosensory imagery have intrinsic value to efforts aimed at restoring sensation in injured people. However, it is likely that there would be a high degree of correspondence between the neural representation of sensations during somatosensory imagery and actual somatosensation (Hodge et al., 1996; Rosén et al., 2001; Yoo et al., 2003; Fitzgibbon et al., 2012). As seen in the motor system (Jeannerod, 1994; Hardwick et al., 2018), research into motor control, motor learning and motor BMIs have shown a high degree of similarity between the neural activity of imagined and executed behavior.

In the longitudinal comparison of the neural representation of sensation (Fig. 4), classification accuracy decreased in most phases and locations compared with testing within the experiments (Fig. 2), with the biggest decrease in performance observed in S1. While it is unclear what caused this change in classification accuracy, it is interesting to note that it was accompanied by the participant's comments during experiment 2 that the passage of time between the two experiments “made it much harder to imagine the sensation [evoked by ICMS] because I have not felt them in a while.” This anecdotal evidence might suggest a link between the strength of responses in S1 to the clarity with which the sensations could be recalled, as may be intuitively expected in a somatosensory imagery task. Nevertheless, threshold crossing S1 activity was still able to yield significant longitudinal classification accuracy after 11 months, comparable to that measured initially. In SMG, the presence of significant classification across experiments may suggest a stronger representation of the task than PMv or S1. The cross-classification performance across the two experiments suggests that while each of these areas encode the sensations after 11 months, the representation over all brain areas differs over time. While physiological changes in the representation of the sensations or the quality of the imagery could contribute to this, there are many additional factors unrelated to the neurophysiology of the task that likely contribute as well. For example, small movements in the array, degradation of the array over time and changes at the electrode-tissue interface may all account for the reduced performance.

Identifying a stable relationship between aspects of the neural signal representing sensations during somatosensory imagery and features of stimulation that evoke those sensations could allow us to efficiently identify protocols for artificially eliciting sensation. This is relevant to closed loop BMIs where during robotic or computer control, task-relevant sensations must be identified and delivered via ICMS. It remains to be investigated whether correspondence between features of the neural signal during imagery and the neural signal evoked during stimulation could reduce the time to map the relationship between sensations and stimulation. If so, somatosensory imagery could be used to improve sensory mapping by stimulation and potentially elicit more varied responses in future work. Furthermore, S1 was originally chosen as a stimulation site because of its known neurophysiological relationship to sensation. Here, we confirm a relationship between imagined sensations and S1 neurophysiology for sensations previously elicited with S1 stimulation in the same array. Somatosensory imagery of the sensations shows an even stronger relationship between neurophysiological activity and imagined sensation in SMG. Therefore, SMG may also be a potential target for ICMS to elicit sensation. Stimulation in parietal cortex has previously been shown to have connections with (Baldwin et al., 2017) and relate to behavior of (Hanks et al., 2006; Mirpour et al., 2010; Desmurget et al., 2018) the sensorimotor system.

In conclusion, we present evidence that human somatosensory imagery can be uniquely and robustly encoded in the activity of distributed cortical areas. In future work it would be essential to identify the evoked neurophysiology from certain stimulation parameters and compare this, instead of stimulation parameters alone, to the evoked sensations and representation of the sensations during imagery or experience. Such information would likely elucidate further the relationship between the stimulation parameters, their ability to elicit certain sensations, and the representation of the sensations elicited in the brain.

## References

[B1] Aflalo T, Kellis S, Klaes C, Lee B, Shi Y, Pejsa K, Shanfield K, Hayes-Jackson S, Aisen M, Heck C, Liu C, Andersen RA (2015) Decoding motor imagery from the posterior parietal cortex of a tetraplegic human. Science 348:906–910. 10.1126/science.aaa5417 25999506 PMC4896830

[B2] Andersen RA, Buneo CA (2002) Intentional maps in posterior parietal cortex. Annu Rev Neurosci 25:189–220. 10.1146/annurev.neuro.25.112701.142922 12052908

[B3] Baldwin MKL, Cooke DF, Krubitzer L (2017) Intracortical microstimulation maps of motor, somatosensory, and posterior parietal cortex in tree shrews (*Tupaia belangeri*) reveal complex movement representations. Cereb Cortex 27:1439–1456. 10.1093/cercor/bhv329 26759478 PMC6075024

[B4] Baumann MA, Fluet M-C, Scherberger H (2009) Context-specific grasp movement representation in the macaque anterior intraparietal area. J Neurosci 29:6436–6448. 10.1523/JNEUROSCI.5479-08.2009 19458215 PMC6665886

[B5] Binkofski F, Dohle C, Posse S, Stephan KM, Hefter H, Seitz RJ, Freund HJ (1998) Human anterior intraparietal area subserves prehension: a combined lesion and functional MRI activation study. Neurology 50:1253–1259. 10.1212/wnl.50.5.1253 9595971

[B6] Callier T, Brantly NW, Caravelli A, Bensmaia SJ (2020) The frequency of cortical microstimulation shapes artificial touch. Proc Natl Acad Sci USA 117:1191–1200. 31879342 10.1073/pnas.1916453117 PMC6969512

[B7] Canolty RT, Edwards E, Dalal SS, Soltani M, Nagarajan SS, Kirsch HE, Berger MS, Barbaro NM, Knight RT (2006) High gamma power is phase-locked to theta oscillations in human neocortex. Science 313:1626–1628. 10.1126/science.1128115 16973878 PMC2628289

[B8] Christie BP, Tat DM, Irwin ZT, Gilja V, Nuyujukian P, Foster JD, Ryu SI, Shenoy KV, Thompson DE, Chestek CA (2015) Comparison of spike sorting and thresholding of voltage waveforms for intracortical brain–machine interface performance. J Neural Eng 12:016009. 10.1088/1741-2560/12/1/016009 25504690 PMC4332592

[B9] Culham JC, Danckert SL, Souza JFXD, Gati JS, Menon RS, Goodale MA (2003) Visually guided grasping produces fMRI activation in dorsal but not ventral stream brain areas. Exp Brain Res 153:180–189. 10.1007/s00221-003-1591-5 12961051

[B10] Dai J, Zhang P, Sun H, Qiao X, Zhao Y, Ma J, Li S, Zhou J, Wang C (2019) Reliability of motor and sensory neural decoding by threshold crossings for intracortical brain–machine interface. J Neural Eng 16:036011. 10.1088/1741-2552/ab0bfb 30822756

[B11] Delhaye BP, Long KH, Bensmaia SJ (2018) Neural basis of touch and proprioception in primate cortex. Compr Physiol 8:1575–1602. 10.1002/cphy.c170033 30215864 PMC6330897

[B12] Desmurget M, Richard N, Beuriat P-A, Szathmari A, Mottolese C, Duhamel J-R, Sirigu A (2018) Selective inhibition of volitional hand movements after stimulation of the dorsoposterior parietal cortex in humans. Curr Biol 28:3303–3309.e3. 10.1016/j.cub.2018.08.027 30318348

[B13] Dong WK, Chudler EH, Sugiyama K, Roberts VJ, Hayashi T (1994) Somatosensory, multisensory, and task-related neurons in cortical area 7b (PF) of unanesthetized monkeys. J Neurophysiol 72:542–564. 10.1152/jn.1994.72.2.542 7983518

[B14] Fitzgibbon BM, Enticott PG, Rich AN, Giummarra MJ, Georgiou-Karistianis N, Bradshaw JL (2012) Mirror-sensory synaesthesia: exploring 'shared' sensory experiences as synaesthesia. Neurosci Biobehav Rev 36:645–657. 10.1016/j.neubiorev.2011.09.006 21986634

[B15] Flesher SN, Collinger JL, Foldes ST, Weiss JM, Downey JE, Tyler-Kabara EC, Bensmaia SJ, Schwartz AB, Boninger ML, Gaunt RA (2016) Intracortical microstimulation of human somatosensory cortex. Sci Transl Med 8:361ra141. 10.1126/scitranslmed.aaf8083 27738096

[B16] Fogassi L, Gallese V, Fadiga L, Luppino G, Matelli M, Rizzolatti G (1996) Coding of peripersonal space in inferior premotor cortex (area F4). J Neurophysiol 76:141–157. 10.1152/jn.1996.76.1.141 8836215

[B17] Graziano MSA (1999) Where is my arm? The relative role of vision and proprioception in the neuronal representation of limb position. Proc Natl Acad Sci USA 96:10418–10421. 10.1073/pnas.96.18.10418 10468623 PMC17903

[B18] Graziano MSA, Hu XT, Gross CG (1997) Visuospatial properties of ventral premotor cortex. J Neurophysiol 77:2268–2292. 10.1152/jn.1997.77.5.2268 9163357

[B19] Gregoriou GG, Borra E, Matelli M, Luppino G (2006) Architectonic organization of the inferior parietal convexity of the macaque monkey. J Comp Neurol 496:422–451. 10.1002/cne.20933 16566007

[B20] Hanks TD, Ditterich J, Shadlen MN (2006) Microstimulation of macaque area LIP affects decision-making in a motion discrimination task. Nat Neurosci 9:682–689. 10.1038/nn1683 16604069 PMC2770004

[B21] Hardwick RM, Caspers S, Eickhoff SB, Swinnen SP (2018) Neural correlates of action: comparing meta-analyses of imagery, observation, and execution. Neurosci Biobehav Rev 94:31–44. 10.1016/j.neubiorev.2018.08.003 30098990

[B22] Hodge C, Dubroff J, Huckins S, Szeverenyi N (1996) Somatosensory imagery activates primary sensory cortex in human: a functional MRI study. Neuroimage 3:S209. 10.1016/S1053-8119(96)80211-4

[B23] Hyvärinen J, Poranen A (1978) Receptive field integration and submodality convergence in the hand area of the post-central gyrus of the alert monkey. J Physiol 283:539–556. 10.1113/jphysiol.1978.sp012518 102768 PMC1282794

[B24] Iwamura Y, Tanaka M, Sakamoto M, Hikosaka O (1993) Rostrocaudal gradients in the neuronal receptive field complexity in the finger region of the alert monkey's postcentral gyrus. Exp Brain Res 92:360–368. 10.1007/BF00229023 8454001

[B25] Jeannerod M (1994) The representing brain: neural correlates of motor intention and imagery. Behav Brain Sci 17:187–202. 10.1017/S0140525X00034026

[B26] Kim S, Callier T, Tabot GA, Gaunt RA, Tenore FV, Bensmaia SJ (2015a) Behavioral assessment of sensitivity to intracortical microstimulation of primate somatosensory cortex. Proc Natl Acad Sci USA 112:15202–15207. 10.1073/pnas.1509265112 26504211 PMC4679002

[B27] Kim S, Callier T, Tabot GA, Tenore FV, Bensmaia SJ (2015b) Sensitivity to microstimulation of somatosensory cortex distributed over multiple electrodes. Front Syst Neurosci 9:47. 25914630 10.3389/fnsys.2015.00047 PMC4392613

[B28] Leinonen L, Hyvärinen J, Nyman G, Linnankoski I (1979) I. Functional properties of neurons in lateral part of associative area 7 in awake monkeys. Exp Brain Res 34:299–320. 10.1007/BF00235675 105918

[B29] Mirpour K, Ong WS, Bisley JW (2010) Microstimulation of posterior parietal cortex biases the selection of eye movement goals during search. J Neurophysiol 104:3021–3028. 10.1152/jn.00397.2010 20861428 PMC3007667

[B30] Neal JW, Pearson RCA, Powell TPS (1990) The ipsilateral cortico-cortical connections of area 7b, PF, in the parietal and tempral lobes of the monkey. Brain Res 524:119–132. 10.1016/0006-8993(90)90500-B 1698108

[B31] Oby ER, Perel S, Sadtler PT, Ruff DA, Mischel JL, Montez DF, Cohen MR, Batista AP, Chase SM (2016) Extracellular voltage threshold settings can be tuned for optimal encoding of movement and stimulus parameters. J Neural Eng 13:036009. 10.1088/1741-2560/13/3/036009 27097901 PMC5931220

[B32] Romo R, de Lafuente V (2013) Conversion of sensory signals into perceptual decisions. Prog Neurobiol 103:41–75. 10.1016/j.pneurobio.2012.03.007 22472964

[B33] Romo R, Hernández A, Zainos A, Salinas E (1998) Somatosensory discrimination based on cortical microstimulation. Nature 392:387–390. 10.1038/32891 9537321

[B34] Rosén G, Hugdahl K, Ersland L, Lundervold A, Smievoll AI, Barndon R, Sundberg H, Thomsen T, Roscher BE, Tjølsen A, Engelsen B (2001) Different brain areas activated during imagery of painful and non-painful 'finger movements' in a subject with an amputated arm. Neurocase 7:255–260. 10.1093/neucas/7.3.255 11459920

[B35] Salas MA, Bashford L, Kellis S, Jafari M, Jo H, Kramer D, Shanfield K, Pejsa K, Lee B, Liu CY, Andersen RA (2018) Proprioceptive and cutaneous sensations in humans elicited by intracortical microstimulation. Elife 7:e32904. 10.7554/eLife.32904 29633714 PMC5896877

[B36] Seelke AMH, Padberg JJ, Disbrow E, Purnell SM, Recanzone G, Krubitzer L (2012) Topographic maps within Brodmann's area 5 of macaque monkeys. Cereb Cortex 22:1834–1850. 10.1093/cercor/bhr257 21955920 PMC3388892

[B37] Sombeck JT, Miller LE (2019) Short reaction times in response to multi-electrode intracortical microstimulation may provide a basis for rapid movement-related feedback. J Neural Eng 17:016013. 10.1088/1741-2552/ab5cf3 31778982 PMC7189902

[B38] Taoka M, Toda T, Iriki A, Tanaka M, Iwamura Y (2000) Bilateral receptive field neurons in the hindlimb region of the postcentral somatosensory cortex in awake macaque monkeys. Exp Brain Res 134:139–146. 10.1007/s002210000464 11037280

[B39] Yoo SS, Freeman DK, McCarthy JJI, Jolesz FA (2003) Neural substrates of tactile imagery: a functional MRI study. Neuroreport 14:581–585. 12657890 10.1097/00001756-200303240-00011

[B40] Zhang CY, Aflalo T, Revechkis B, Rosario ER, Ouellette D, Pouratian N, Andersen RA (2017) Partially mixed selectivity in human posterior parietal association cortex. Neuron 95:697–708.e4. 10.1016/j.neuron.2017.06.040 28735750 PMC5572762

